# Measuring relational memory in older and younger adults

**DOI:** 10.3389/fcogn.2025.1499032

**Published:** 2025-02-18

**Authors:** Jennifer N. Sexton, Lillian Behm, Jill A. Rose, Connor J. Phipps, Meghan K. Ramirez, Abi M. Heller-Wight, Anna F. Wilhelm, Emma A. Armbruster, Carolyn E. Nagengast, David E. Warren

**Affiliations:** Department of Neurological Science, University of Nebraska Medical Center, Omaha, NE, United States

**Keywords:** episodic memory, relational memory, associative memory, item memory, aging

## Abstract

**Introduction:**

Changes in cognitive abilities including memory accompany normal aging, and certain types of memory are particularly susceptible to age-related change. The ability to link aspects of an experience to form one cohesive memory, called relational memory, is essential to normal memory throughout the lifespan. Relational memory facilitates the binding of arbitrarily related stimuli and encompasses all manner of relations (spatial, associative, sequential). Prior work has studied differences in relational memory associated with aging but has investigated specific aspects of relational memory in a siloed fashion: earlier studies typically have not simultaneously assessed multiple aspects of relational memory in the same participants in the same paradigm.

**Methods:**

In the current study, multiple aspects of relational memory were simultaneously compared between healthy younger adults (19–35 years, *n* = 40) and healthy older adults (65–77 years, *n* = 40).

**Results:**

We found that older adults had reduced memory performance relative to younger adults on each condition of the memory task (item condition, space condition, re-pair condition, and time condition), and there was a condition-by-age group interaction such that differences were greatest for the time and space conditions.

**Discussion:**

We found age-related differences between young and older adults on a task simultaneously testing multiple types of relational memory with young adults performing better overall. Additionally, we observed condition-level interactions such that the age-related differences were greater for the time and space conditions than the re-pair condition. Together, these findings underscore the importance of measuring memory for all manner of relations using the same study format to achieve a thorough characterization of the complex nuances of relational memory performance across the lifespan.

## Introduction

Key goals for memory research are understanding how stimuli are encoded, stored, and retrieved, and a special case of these processes involves situations when two or more stimuli are presented together. For example, remembering the name of a new colleague upon first meeting her requires memory for her name, her face, and the relation between the two. Researchers have described the process of remembering associations between stimuli using descriptors such as “relational memory” (Konkel and Cohen, 2009) as described by relational memory theory (Eichenbaum and Cohen, 2001), “associative memory” based on the processing-based memory model (Henke, 2010), and more broadly as a component of “episodic memory” (Shing et al., 2010). Relational memory, associative memory, and episodic memory are similar in that they emphasize the binding together of items that comprise an experience, but relational memory theory emphasizes a key structure-function association by suggesting that the hippocampus is necessary for encoding durable memories of arbitrary relations among items. So while the processing-based model is an extension of relational memory theory (as reviewed in Henke, 2010), relational memory theory distinguishes memory for items from memory for relations between items and proposes the essential role of the hippocampus for relations (Cohen and Eichenbaum, 1993; Eichenbaum and Cohen, 2001). Critically, variability in descriptive terminology and measurement methodology for putatively different types of memory (associative vs. relational) or different populations (younger adults vs. older adults) may unintentionally impose a horizontal limitation within the field of memory research, potentially impeding progress toward understanding the underlying memory processes and the contributing brain systems.

Described as a cognitive process, relational memory supports memory for all manner of relations between stimuli. It facilitates memory for the co-occurrence of items presented together (associative relations), how things are positioned relative to one another in space (spatial relations), and the relative timing or order of stimuli (temporal relations). Relational memory can be constrasted with item memory, which supports memory for individual items. Everyday memory relies on relational memory processes: when you remember where you parked your car in a full parking lot you must remember how your car relates to other landmarks in the environment (spatial relations); when you see a familiar person in public and remember they are a colleague in your research department (associative relations); or when you have separate plans with two friends and must remember whom you are meeting with first (temporal relations).

Deficits in relational memory may be particularly detrimental to memory for episodes because relational memory links otherwise arbitrarily related elements (e.g., person, place, time) of an experience to form one cohesive memory (Naveh-Benjamin et al., 2003). Deficits in relational memory throughout aging (sometimes described as the associative deficit hypothesis; Naveh-Benjamin, 2000) have been reported in earlier work both in healthy aging people (Bender et al., 2010) and in people with age-related neuropathologies (Lowndes and Savage, 2007). These changes in memory have been associated with structural and functional changes in the brain. One region of particular interest that appears to contribute to age-related cognitive changes in memory is the hippocampus (Eichenbaum et al., 1999). The hippocampus is necessary for normal relational memory, and changes in the hippocampus are observed during healthy aging (Fjell et al., 2014).

Previous literature supports age-related differences in relational memory, such that older adults show worse relational memory than younger adults (Naveh-Benjamin, 2000), but memory for certain types of relations may be more affected by age than others. A meta-analysis from Old and Naveh-Benjamin found that age effects are quite pronounced for studies implementing paradigms measuring temporal relations and spatial relations (Old and Naveh-Benjamin, 2008). In contrast, they found that while age-related effects were still prominent in studies that measured associations between item pairs, the effects tended to be smaller. More recent studies also lend support for testing different relational memory types when describing age-related changes in memory. For instance, in a study comparing older and younger adults on a spatial binding task, the age groups did not show significant differences in accuracy during retrieval, despite younger adults showing a faster response time (Rondina et al., 2019). This can be compared to results found by Endemann and Kamp, 2022, where older and younger adults showed robust, age-related differences in memory for associative relations when asked to remember pairs of items or images.

Variability in neuropsychological assessments and experimental paradigms to probe relational memory may account for discrepancies across studies regarding the extent to which memory for specific types of relations is differently affected by aging. Previous studies have used context-dependent relational memory tasks (Schwarb et al., 2015), manipulated scene tasks (Hannula et al., 2015), reconstruction of object array tasks (Watson et al., 2013), item/word pairing tasks (Endemann and Kamp, 2022; Hugeri et al., 2022; Ngo et al., 2019; Naveh-Benjamin, 2000; Naveh-Benjamin et al., 2003), person-object-location triplets (Joensen et al., 2024), and multiple source information (Uncapher et al., 2006). These tasks rely on memory for items, item pairs/co-occurrence of items, spatial relationships, or temporal relationships. These studies describe their respective paradigms as measuring relational memory (Schwarb et al., 2015; Hannula et al., 2015; Watson et al., 2013), associative memory (Endemann and Kamp, 2022; Hugeri et al., 2022; Naveh-Benjamin, 2000; Naveh-Benjamin et al., 2003), and episodic memory (Ngo et al., 2019; Joensen et al., 2024; Uncapher et al., 2006).

As illustrated in the above studies, the diverse terminology utilized within the memory literature can create challenges for interpretation, hindering synthesis within the field of memory. In addition, targeted findings from previous literature in this domain have most often studied relations between items in a siloed fashion, measuring memory for one type of relation at a time using distinct stimuli and paradigms, thereby limiting the potential for comparison within subjects. Comparing memory for different types of relations using a within-subjects design might reveal specific differences in relational memory components between younger and older adults. Furthermore, understanding these differences could inform the development of targeted interventions to preserve or even enhance memory in healthy older adults by mitigating effects of age-related cognitive decline in memory.

This gap in the literature could be filled by a memory task that allows simultaneous measurement of memory for two or more types of relations. To address this need, a computer-based relational memory task, called the All Manner of Relations (AMR) task, was developed to measure several distinct forms of relational memory (Konkel et al., 2008). The AMR task differs from other tasks in that it specifically tests multiple types of relational memory, one at a time, using similar novel visual stimuli presented in the same manner in the same session and within subjects. In doing so, the AMR task aims to surmount barriers in prior work studying relational/associative memory by supporting measurement of memory for temporal relations, spatial relations, and associative relations using the same stimuli and study format. Investigating memory for multiple types of relations contemporaneously within subjects could provide insight regarding age-related differences in relational memory that would not be possible if the different domains of memory were tested separately and/or between subjects.

The AMR task assesses memory for spatial, associative, and sequential relations as well as item memory. Spatial relations represent the association between items in a space; associative relations represent the association between objects when grouped; and sequential relations represent the association between items in time. A participant's performance on these tasks can be compared to their item memory which represents memory for previously encoded items. This task has been used to assess relational memory in patients with memory deficits following hippocampal damage (Konkel et al., 2008) and was also adapted for studies of relational memory in children (Lee et al., 2016, 2020; Pathman et al., 2018), but it has yet to be utilized with healthy younger or older adults.

In the current study, we set out to determine the generalizability of the AMR task for measuring relational memory in older and younger adults. For the AMR task to be generalizable to older and younger adult samples, it must replicate previous findings on age differences in item memory and relational memory performance. We hypothesized that older adults would perform less well on the AMR task than younger adults on all task conditions, with performance on the item condition being the most similar between the two age groups. The aim of the present study was to extend knowledge regarding age-related differences in relational memory performance by addressing all manner of relations with the same stimuli in the same sample, and in doing so, determine whether the AMR task could generalize to healthy young and older adults.

## Materials and methods

### Participants

We enrolled young adults (age range: 19–35 years) and older adults (age range: 65–77 years) from the Omaha, Nebraska community. Participants were a convenience sample from the Omaha community, and data were collected at the University of Nebraska Medical Center (UNMC). Participants were recruited as part of a study that had the following exclusionary criteria: left-handedness, neurological disease, medications for major medical illness, and being a non-native English speaker. The total sample included 40 younger adults (26 females), (mean age = 25.1 years, *SD* = 4.0 years), and 40 older adults (24 females), (mean age = 71.2 years, *SD* = 3.1 years). Collectively, participant ages ranged from 19 to 77 years (*M* = 48.2 years, *SD* = 23.3) (nb. the age of our adult sample was ≥19 years because Nebraska's age of majority is 19 years). Demographic information was collected for all participants and data on educational attainment was collected for the older adult sample. See Table 1 for demographic information and distribution of age by sex. All protocol and procedures employed in this study were approved prior to the start of the study by the UNMC Institutional Review Board. Informed consent was obtained from all participants prior to data collection. Participants were remunerated for their time.

**Table 1:** Descriptive statistics for age, sex, and education.

**Variable**	***N***		**All**	**Males**	**Females**
			***M*** **(*****SD*****)**	***M*** **(*****SD*****)**	***M*** **(*****SD*****)**
**Age (years)**					
Younger adults	40 (26 F)		25.10 (4.08)	25.57 (4.34)	24.85 (3.87)
Older adults	40 (24 F)		71.20 (3.09)	71.13 (2.66)	71.25 (3.40)
**Education (older adults)**					
High school or less	Some college	Associate's	Bachelor's	Master's	Professional/ Doctoral
3 (7%)	6 (15%)	2 (5%)	19 (48%)	8 (20%)	2 (5%)

*N* = 80.

### Procedure

**Figure 1:**
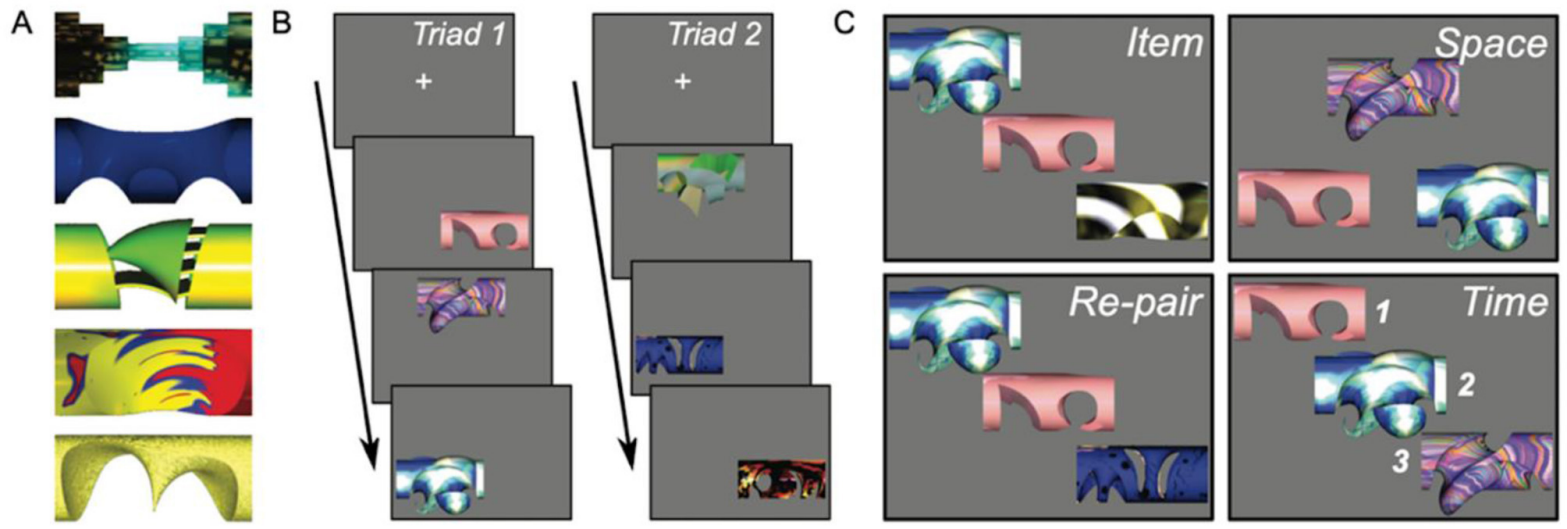
All manner of relational memory task. The figure demonstrates the All Manner of Relations (AMR) task. **(A)** shows example arbitrary stimuli used in the AMR task. **(B)** demonstrates the study phase when triads of stimuli are demonstrated to participants. Each stimulus is shown in a set of three in a particular temporal order and distinct spatial arrangement on the screen. **(C)** demonstrates the testing phase, which occurs after the study phase. At the end of the study phase, participants are tested for memory. Participants memory was tested for each item and spatial, associative, and sequential relations between items.

In the AMR task, participants underwent several rounds of study-test phases (six study-test phases were completed per task condition). Regardless of the subsequent condition tested, each study phase was the same apart from the orienting encoding instructions. In the study phase, participants were presented with three stimuli (a “triad”) in a sequential fashion (1st, 2nd, 3rd), each in one of three unique positions on the screen (lower left, lower right, upper center). Each stimulus was presented in isolation on the screen without competing information. Prior to each block, participants were instructed to remember either the items (item memory) or a specific relation between the items (relational memory; see Figure 1). For item memory, participants were asked to remember each stimulus presented to them (the “item” condition testing item memory), but not necessarily the association of these items together in a triad. In each relational memory condition, participants were instructed to remember how the stimuli were associated with the other stimuli presented either in location on the screen (the “space” condition testing spatial relations), in order of presentation (the “time” condition testing temporal relations), or with which other stimuli they were presented (the “re-pair” condition testing associative relations). Importantly, the re-pair condition and the item memory condition differ in that the former requires participants to remember which items were assigned together as a triad previously, whereas the latter only requires the participants to remember that they saw each of the items previously, whether or not they belonged together in a triad. The isolated presentation of each stimulus of course occurs in a spatial and temporal context, but our protocol balanced relations of various types in a way that supported rigorous testing of item memory, spatial relations, associative relations, and temporal relations. Each participant was asked to remember all four conditions in separate blocks throughout the task. Each triad was separated from the preceding and subsequent triads by a fixation cross. Triads were presented on the screen for an interval of 3 seconds, followed by a 2-s fixation cross. Each study phase included three sets of triads, each presented twice non-consecutively to allow sufficient opportunity for encoding. The stimuli used were intraexperimentally novel and unfamiliar to the participants, thus reducing likelihood of verbal rehearsal while supporting comparison between item memory and three types of relational memory following the same study format.

After each study phase, memory for the studied triads was tested. During the test phase, one type of relational memory was probed at a time to ensure each type of memory was not confounded with other representations. Thus, in separate blocks, memory for items, spatial relations between items, associative relations between items, and sequential relations between items were assessed (Konkel et al., 2008, see Figure 1). During the test phase for the item, space, and re-pair conditions, triads were presented on the screen simultaneously for 6 s, compared to the sequential presentation during the study phase. Participants were instructed to respond “yes” if the stimuli or target relation matched those presented at study or “no” if the stimuli or target relation did not match those presented at study. After 6 s, the test screen was replaced with a statement reminding participant to respond, and then they had as long as necessary to respond. For the time condition, participants were instructed to respond as in other conditions, but they were shown the stimuli from a studied triad one at a time for 2 s each to allow for a test of temporal relations. This was followed by a test screen instructing them to respond which persisted until a response was given. Breaks were given between study-test blocks as needed.

Test phases contained either repeated study triads (repeated trial) or manipulated sets (manipulated trial). For the item memory test phase, repeated trials contained previously studied triads whereas manipulated trials contained two studied items and one non-studied item. For the space condition, repeated trials contained previously studied triads in their original location, whereas manipulated trials contained previously studied triads with two of the item locations swapped. For the re-pair condition, repeated trials contained previously studied triads that belonged together, whereas the manipulated trials contained previously studied stimuli that did not belong to the same triad. For the time condition, repeated trials contained previously studied triads in their original order, whereas manipulated trials contained previously studied triads with the order of two of the stimuli switched. Each test phase contained 9 repeated trials and 9 manipulated trials.

Counterbalancing was conducted by varying the groups of items presented by condition (stimulus set A, stimulus set B, stimulus set C, stimulus set D), and by counterbalancing task condition order (item condition, space condition, re-pair condition, and time condition). Each stimulus set was either always studied together or not studied. Non-studied stimuli were available as novel lures for manipulated trials in the item condition. These counterbalancing conditions were independent and orthogonal, leading to 16 possible permutations. For more information on the design of the AMR task, see (Konkel et al., 2008).

### Analyses

Memory performance was quantified using measures adapted from signal detection theory (SDT; Green and Swets, 1966). A trial where the participant correctly identified a triad that was studied together in the test phase was labeled a hit. Alternatively, a trial where the participant falsely identified a triad that was not presented together was labeled a false alarm. When a participant did not correctly identify a triad that was presented together, it was labeled as a miss, and when a participant correctly identified items that did not belong to the same studied group, it was labeled a correct rejection. The SDT-derived sensitivity index, d', reflects the normalized hit rate minus the normalized false alarm rate and was used in this study to measure relational memory task performance. The SDT metrics are valuable for examining memory in older adults as they enable the measurement of memory sensitivity. This may be especially important in the context of studies comparing younger and older adults because older adults have increased susceptibility to false memories (for a review, see Devitt and Schacter, 2016).

A two-way mixed factors ANOVA was conducted to compare the effects of age group (between-subject variable), AMR condition (within-subject variable), and the interaction of age and AMR condition on relational memory performance as measured by the sensitivity index for item, space, re-pair, and time conditions. The ANOVA test was followed by pairwise comparisons and planned comparisons between groups overall and for each condition, implemented as non-paired, equal-variance *t*-tests. The magnitude of difference in performance between age groups by task condition was compared using the Stieger's Z test (Steiger, 2004). A one-sample *t*-test per group/condition was also conducted to assess whether performance on the AMR task was above-chance levels for both age groups across all conditions. The effects of potentially confounding variables, including educational attainment, sex, and counterbalancing condition were investigated using simple linear regression. Statistical analysis was completed in R (version 4.1.2) and was visualized in both R (version 4.1.2) and IBM SPSS Statistics (Version 29.0.0.0).

## Results

**Figure 2:**
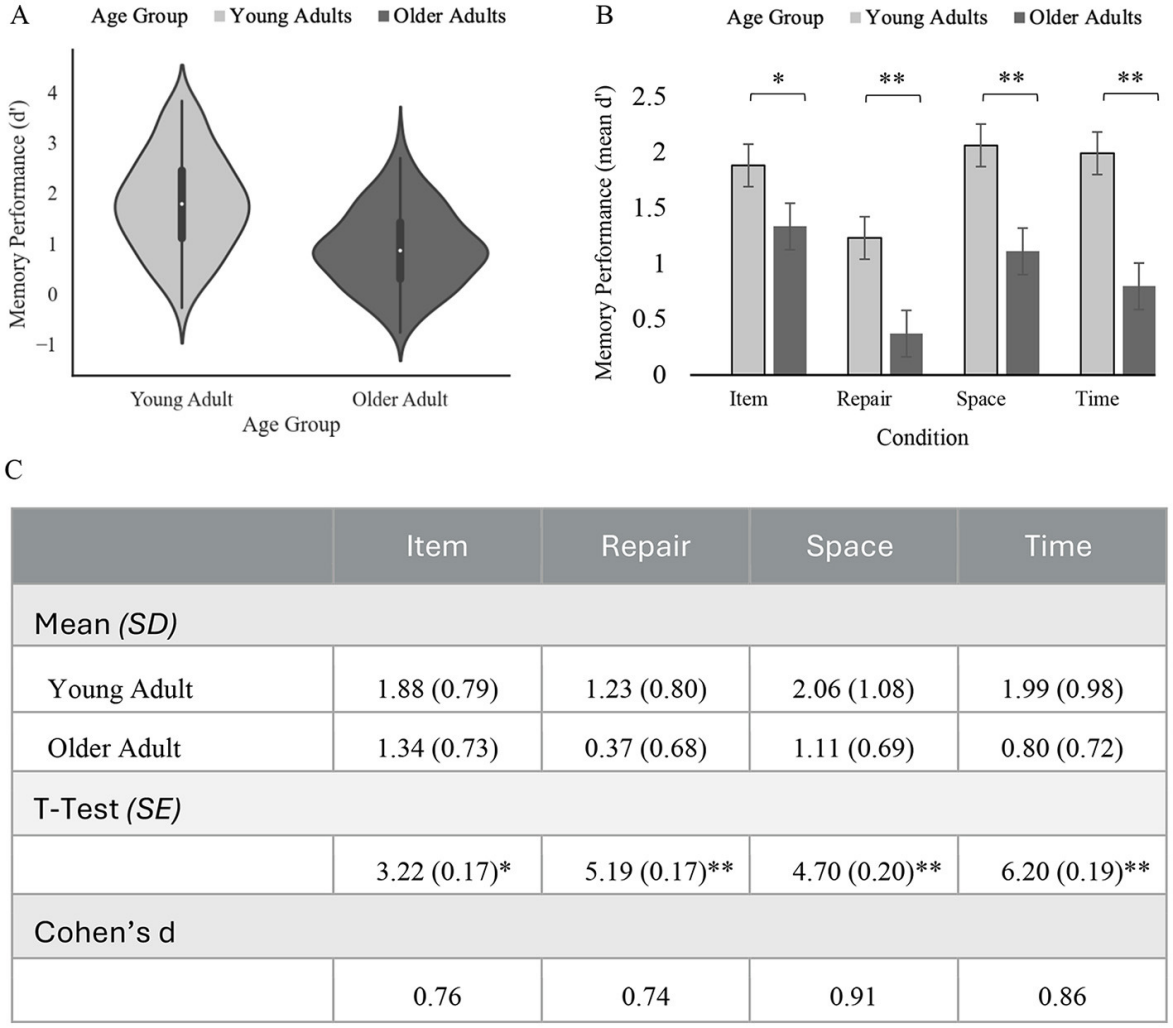
Memory performance by age group and condition. **(A)** displays a violin plot of memory performance (d') across all task conditions measured by performance between age groups. **(B)** displays mean memory performance by task condition and age group. Error bars represent ±2 standard error. **(C)** describes mean performance. SD, Standard deviation; SE, Standard Error. ^*^p < 0.01, ^**^p < 0.001.

The analysis of variance showed a main effect of age group on memory performance that was statistically significant, [*F*_(1, 78)_ = 38.91, *p* < 0.001; see Table 2]. Across all memory conditions, performance for older adults (*M* = 0.90, *SD* = 0.51) was significantly lower than younger adults [*M* = 1.79, *SD* = 0.74, *t*_(78)_ = 6.24, *p* < 0.001, *d* = 0.64; see Figure 2A]. Comparing task conditions, younger adults outperformed older adults on the item condition [younger adults: *M* = 1.88, *SD* = 0.79 vs. older adults: *M* = 1.34, *SD* = 0.73; difference in average d' score between age groups = 0.54, *t*_(78)_ = 3.22, *p* = 0.002, *d* = 0.72], space condition [younger adults: *M* = 2.06, *SD* = 1.08 vs. older adults: *M* = 1.11, *SD* = 0.69, difference in average d' score between age groups = 0.95, *t*_(78)_ = 4.70, *p* < 0.001, *d* = 1.05], re-pair condition [younger adults: *M* = 1.23, *SD* = 0.80 vs. older adults: *M* = 0.37, *SD* = 0.68, *t*_(78)_ = 5.19, difference in average d' score between age groups = 0.86, *p* < 0.001, *d* = 1.16], and time condition [older adults: *M* = 0.80, *SD* = 0.72 vs. younger adults: *M* = 1.99, *SD* = 0.98, difference in average d' score between age groups = 1.19, *t*_(78)_ = 6.20, *p* < 0.001, *d* = 1.39, see Figure 2B].

**Table 2:** Analysis of variance of performance.

**Effect**	***df*_1_**	***df*_2_**	***F***	***p***
Age group	1	78	38.91	< 0.001
Condition	3	78	31.94	< 0.001
Age group × condition	3	78	4.02	0.008

*N* = 80.

There was also a significant main effect of AMR condition on memory performance, [*F*_(3, 78)_ = 31.94, *p* < 0.001; see Figures 2B, C]. Across all participants, performance on the item condition (*M* = 1.61, *SD* = 0.81) was significantly greater than performance on the re-pair condition (*M* = 0.80, *SD* = 0.85), *t*_(80)_ = 9.84, *p* < 0.001, and the time condition (*M* = 1.40, *SD* = 1.05), *t*_(80)_ = 2.13, *p* = 0.036, but it was not statistically different than performance on the space condition (*M* = 1.59, *SD* = 1.02), *t*_(80)_ = 0.21, *p* = 0.832. For the total sample, performance on the re-pair condition was significantly less than performance on the time condition, *t*_(80)_ = 6.29, *p* < 0.001, and the space condition, *t*_(80)_ = 7.86, *p* < 0.001. Meanwhile, performance on the time condition was significantly lower than performance on the space condition, *t*_(80)_ = 2.01, *p* = 0.048. On average, all participants performed above chance for all conditions of the task (each T > 3.49, each p < 0.001).

The interaction of AMR condition and age group on memory performance was also significant, [*F*_(3, 78)_ = 4.02, *p* = 0.008], implying that the condition of the task significantly impacted the memory performance differently between the young adults and older adults. When comparing performance by condition between age groups, the difference in performance was largest for the time condition (difference in average d' score between age groups = 1.19), whereas the magnitude of difference was smallest for item memory (difference in average d' score between age groups = 0.54). The magnitude of difference in performance between age groups by task condition was compared using the Stieger's Z test (Steiger, 2004). The magnitude of difference between young and older adults for the re-pair condition was significantly different than the magnitude of difference between the age groups for the time condition, Z = 2.98, *p* < 0.01, for the space condition, Z = 7.21, *p* < 0.01, and for the item condition, Z = 7.91, *p* < 0.01, such that the group difference was less for re-pair than for space, time, or item condition. This interaction remained evident when systematically excluding one condition at a time, except for when excluding the re-pair condition [*F*_(2, 79)_ = 2.80, *p* =0.064].

Follow-up pairwise comparisons revealed that younger adults outperformed older adults on all conditions (item condition: *p* < 0.01, time condition: *p* < 0.001, space condition: *p* < 0.001, re-pair condition: *p* < 0.001, see Figure 2C). Within the younger adult group, pairwise comparisons revealed that performance between conditions were similar, with the only significant differences in performance reflecting that the performance on the re-pair condition (*M* = 1.23, *SD* = 0.80) was significantly lower than all other conditions (item memory: *M* = 1.88, *SD* = 0.79, *p* < 0.001, space condition: *M* = 2.06, *SD* = 1.08, *p* < 0.001, time condition: *M* = 1.99, *SD* = 0.98, *p* < 0.001). Performance on item memory, space, and time conditions did not show significant differences for young adults (all *p*'s > 0.05). Within the older adult group, pairwise comparisons revealed that performance varied greatly between conditions, with significant differences in performance between the item condition (*M* = 1.34, *SD* = 0.73) and the time condition (*M* = 0.80, *SD* = 0.72; *p* < 0.001), and between the item condition and the re-pair condition (*M* = 0.37, *SD* = 0.68; *p* < 0.001). There was not a significant difference between item memory (*M* = 1.34, *SD* = 0.73) and performance on the space condition (*M* = 1.11, *SD* = 0.69, *p* = 0.091). Additionally, older adults' performance on each of the relational memory conditions were significantly different from each other (space condition vs. time condition, *p* < 0.001, space vs. re-pair condition, *p* < 0.001, and re-pair condition vs. time conditions, *p* < 0.001), with older adults showing the lowest performance on the re-pair condition.

Counterbalancing of the task was not significantly associated with task performance, *F*_(1, 78)_ = 0.08, *p* = 0.776, *R*^2^ = 0.001, nor was the sex of the participant significantly associated with task performance, *F*_(1, 78)_ = 2.51, *p* = 0.117, *R*^2^ = 0.031. Finally, educational attainment task performance for the older adults, *F*_(1, 38)_ = 0.14, *p* = 0.711, *R*^2^ = 0.004.

## Discussion

Progress in our understanding of relational/associative memory has been substantial, but it has also been hindered by research designs not supporting measurement of multiple types of relational memory simultaneously or by not adopting a lifespan perspective to memory research. This study aimed to overcome these barriers and test for differences in relational memory in healthy young and older adults as well as assess the generalizability of the AMR task to these populations. We found that overall, both young and older adults in our study performed above chance on average across all conditions, increasing confidence that the AMR task is suitable for healthy young and older adult samples. Our results suggested that that older adults had reduced memory performance relative to younger adults overall and on each condition of the task (item condition, space condition, re-pair condition, and time condition). This finding was not unexpected: prior work has shown deterioration in both item memory and relational memory throughout aging, but with relational memory deficits typically exceeding item memory deficits (for a review, see Dennis and McCormick-Huhn, 2018). The condition of the task also affected performance, with the highest average performance across both groups on the item condition and the lowest average performance across both groups on the re-pair condition. This finding underscores the importance of measuring all manner of relations after the same study format to achieve a thorough characterization of the complex nuances of relational memory performance.

These findings add to the body of literature suggesting that item memory and relational memory may make separate contributions at retrieval (Buchler et al., 2008). Our findings can compared to the study by Buchler et al. in which participants studied word pairs and were later tested on whether they could discriminate intact word pairs from non-intact word pairs that either included previously unstudied words or two items that had been previously studied, but paired with a different word. Buchler and colleagues found that participants in their study could differentiate new vs. old words in word pairs, whether each word was new, and distinguish recombined pairs from original pairings. Triads in our study may be easier for participants to reject in the item memory condition than the re-pair condition because the novel items presented in the item memory condition did not provoke a sense of familiarity, as the stimuli in the re-pair conditions do. Therefore, our findings may reflect the same underlying phenomena as Buchler et al. but in a novel paradigm and using a within-subjects design (Buchler et al., 2008).

There was also a condition by age group interaction, such that the difference between young adults and older adults depended on task condition, with the item memory performance showing the smallest difference between the groups. Overall, these results are consistent with prior work suggesting that older adults perform significantly less well on tests of relational memory compared to younger adults (Rondina et al., 2017), and relational memory may be particularly sensitive to the effects of aging (see Giovanello and Dew, 2015 for a review) with age-related effects appearing larger for temporal and spatial relations than for item memory (Old and Naveh-Benjamin, 2008). Importantly, our study replicated this pattern of findings using unfamiliar visual stimuli presented in the same study format for each test condition, thus equating study time, format, and load for each relational test, a novel contribution. We found differences in relational memory depending on the type of memory condition assessed, which contrasted with the findings by Hugeri et al. (2022) that found similar performance between memory for spatial, temporal, and item memory for older adults. Interestingly, our study also included associative relations, which drove the interaction effect and may explain the difference in our findings. More research is needed to continue examining aspects of relational memory to better understand these distinct but interrelated processes. Better characterizing age-related changes in relational memory may provide insight to the neurological underpinnings of relational memory and/or may highlight opportunities for intervention.

Our study was not without limitations. We relied on a new implementation of the original task (Konkel et al., 2008) by an author on the original publication (DEW), although all elements of the original task were implemented to reproduce it faithfully including utilization of the same unfamilar visual stimuli. The AMR task may still benefit from additional neuropsychological/standardized testing to validate our results. However, overall patterns of performance were broadly aligned with earlier work. A significant difference was found between young and older adults, as has been found in other studies using validated neuropsychological assessments (Old and Naveh-Benjamin, 2008). The study also recruited community dwelling participants so any non-diagnosed conditions were not known. Deficits in relational memory can be related to a variety of pathological conditions but the participants were healthy, community dwelling young and older adults with normative cognitive abilities for age.

By using a paradigm that is known to depend on the hippocampus and testing specific domains of relational memory in older and younger adults, our study may have also measured contributions of different extrahippocampal brain regions that could have varied between age groups. The poorer performance among older adults may be the result of hippocampal deterioration with healthy aging or inefficient compensation by other brain regions. Assessing these speculative accounts would require structural and/or functional neuroimaging data beyond the scope of the current study, but subsequent research could incorporate measures derived from brain imaging. Future research of this kind might also explore performance on this task longitudinally while accounting for brain variables including cortical thickness, hippocampal volume, or neuropathology among other factors.

Our work extends previous research investigating relational memory in heathy young and older adults. Tasks that support simultaneous assessment of multiple aspects of hippocampal-dependent memory remain rare, but our study demonstrates the generalizability of a task with useful design attributes to new populations as well as an anticipated but novel age-related difference in memory performance across several domains, here measured contemporaneously within subjects within the same task. Implementing paradigms such as the AMR task, those that measure different domains of relational memory while holding stimuli and study format constant, has significant potential to foster synthesis within the field of memory research while also advancing our understanding of fundamental properties of memory processes and the brain systems that support them.

Memory research aims to understand how stimuli are encoded, stored, and retrieved; however, previous work has not typically explored more than one type of relational memory at a time. This gap may be due to the constraints based on how relational and associative memory are defined. Task designs that span gaps in current methodology will allow for a more comprehensive understanding of the binding of arbitrarily related information and, more broadly, further our understanding of hippocampal-dependent memory processes.

## Data Availability

The raw data supporting the conclusions of this article will be made available by the authors, without undue reservation.
